# Updated fine-scale location data for Blantyre district collected using a low-cost approach

**DOI:** 10.1038/s41597-025-05605-5

**Published:** 2025-07-22

**Authors:** Chifuniro Baluwa, Patrick Ken Kalonde, Upendo Lisa Mseka, Owen Tsoka, Kingsley Kajanga, Faith Chimphondah, Prince Time Matope Aaron, Francis Chimbalanga, Blessings Chiepa, Clinton Nkolokosa, Latif Ndeketa, Patrick Musicha, Kondwani Charles Jambo, Michelle C. Stanton, James Chirombo

**Affiliations:** 1 Malawi-Liverpool-Wellcome Research Programme, Chipatala Avenue, Blantyre, Malawi; 2 Liverpool School of Tropical Medicine, Pembroke Place, Liverpool, L3 5QA United Kingdom; 3 Malawi University of Business and Applied Science, Chichiri, Blantyre, Malawi; 4 University of Malawi, Chirunga, Zomba, Malawi; 5 Queen Elizabeth Central Hospital, Blantyre, Malawi; 6 University of Liverpool, Liverpool, United Kingdom; 7 Kamuzu University of Health Sciences, Blantyre, Malawi

**Keywords:** Geography, Infectious diseases

## Abstract

Knowing up-to-date geographical location of residential areas is crucial for understanding health-related factors and formulating targeted interventions. However, such data are often unavailable or resource-intensive to collect. This study employed low-cost approaches to map residential areas in Blantyre district, Malawi, which was severely affected by a cholera outbreak that lasted over a year (March 2022 to August 2023). We trained five data collectors using KoBo Toolbox and engaged Health Surveillance Assistants (HSAs) and motorcycle operators for precise location identification. We validated the data by involving key stakeholders from Blantyre District Health Office (DHO) and Malawi-Liverpool-Wellcome Research Programme (MLW). We successfully mapped 764 locations, demonstrating the effectiveness in rapidly mapping both residential and hard-to-reach areas. Associated costs were calculated based on MLW standard rates. This process highlighted the critical importance of accurate geolocation for public health research and interventions. Our study provides valuable spatial data and showcases the feasibility of cost-effective methods for gathering crucial public health information in resource-limited settings, potentially serving as a model for similar efforts globally.

## Background & Summary

Precise geographical data is crucial for targeted public health interventions, especially in resource-limited settings^1–4^, as demonstrated during door-to-door cholera vaccination campaigns during disease outbreaks^5–7^. Blantyre district in Southern Malawi exemplifies this need. The district was the epicentre of the deadliest cholera outbreak in Malawi, lasting from March 2022 to August 2023, with over two-thirds of the 8,932 confirmed cases and 227 deaths occurring in Blantyre urban alone^8^. The outbreak’s case fatality rate exceeded the World Health Organization (WHO) acceptable threshold of 1%, revealing critical weaknesses in the ability to rapidly identify and reach affected communities, highlighting the urgent need for accurate geospatial data to enable timely and targeted public health response. Despite its importance, such data were lacking, particularly for residential areas reported by cholera patients.

Blantyre district covers approximately 2,012 km^2^, is administratively subdivided into city and rural areas. According to 2023 population projections by the Malawi National Statistical Office (NSO), Blantyre district has an estimated population of 1.38 million, with Blantyre city accounting for about 871,800 inhabitants^9^. Despite the wealth of health records in facilities across Blantyre district, we observed that approximately 60–70% of documented residential areas lack proper mapping and geographical coordinates in open spatial datasets. Our comparison between gazetted residential areas from the district council and areas documented in cholera line listings confirmed this gap. This deficiency stems from several factors: rapid urbanization outpacing formal mapping efforts, limited resources for systematic data collection, informal naming conventions that vary between communities and official records, and the absence of standardized location recording procedures in health facilities^10,11^. Additionally, the proliferation of informal settlements, particularly in urban areas, has created residential locations that exist outside formal administrative recognition. This situation poses significant challenges to understand disease patterns and implement targeted responses based solely on formal administrative boundaries. Targeted public health interventions such as vaccination campaigns require the most accurate and up-to-date geospatial data to optimize limited resources such as cholera vaccine. The absence of up-to-date residential area data presented a major barrier to effective surveillance, outbreak response, and equitable resource allocation, underscoring the critical need to close this geospatial data gap.

Several previous mapping initiatives have attempted to address geolocation challenges in Malawi and similar resource-limited settings. The Malawi Spatial Data Platform (MASDAP)^12^ has served as a central repository for geospatial data, but its coverage of residential areas remains incomplete, particularly in rapidly changing urban environments. Other efforts include OpenStreetMap (OSM) based mapping projects^13^ and initiatives by international organizations such as UNICEF and WHO^14,15^. However, these approaches often rely on expensive equipment, external expertise, or fail to capture informal settlements and rapidly evolving residential areas. While existing technologies such as Geographical Information Systems (GIS), Global Positioning Systems (GPS), and Health Information Systems (HIS) offer promising solutions to address these gaps^16–18^, their implementation in resource-constrained settings like Blantyre has been limited by cost and technical capacity.

Our study addresses these challenges through a novel approach that differentiates itself from existing efforts in three key ways. First, we employ a low-cost, community-embedded methodology that leverages local knowledge through Health Surveillance Assistants (HSAs), dramatically reducing resource requirements compared to conventional mapping exercises. Second, our approach integrates multiple data sources, including real-time cholera outbreak data, enhancing the relevance for immediate public health interventions. Third, our participatory mapping strategy ensures community ownership and sustainability, addressing a critical gap in previous top-down mapping initiatives. By combining these elements, we demonstrate that comprehensive geospatial data collection is feasible even in resource-constrained settings during a public health emergency. Apart from guiding targeted disease control, this data can be used for other applications including helping to guide patient’s residential locations in applications such as electronic participant locator (ePAL)^19,20^.

In Blantyre, health records typically identify disease transmission locations by residential area names rather than precise addresses. However, databases containing these locations are often incomplete, outdated, or entirely missing^21^. This situation is exacerbated by factors such as rapid urban expansion, the proliferation of informal settlements, and resource constraints in data collection^18,22–24^. Our study aimed to address these challenges by generating an updated and geolocated dataset of residential areas in Blantyre district. We used novel and low-cost approach to map both formal and informal settlements, focusing particularly on areas with the least geolocation data available in existing datasets.

**Fig. 1:**
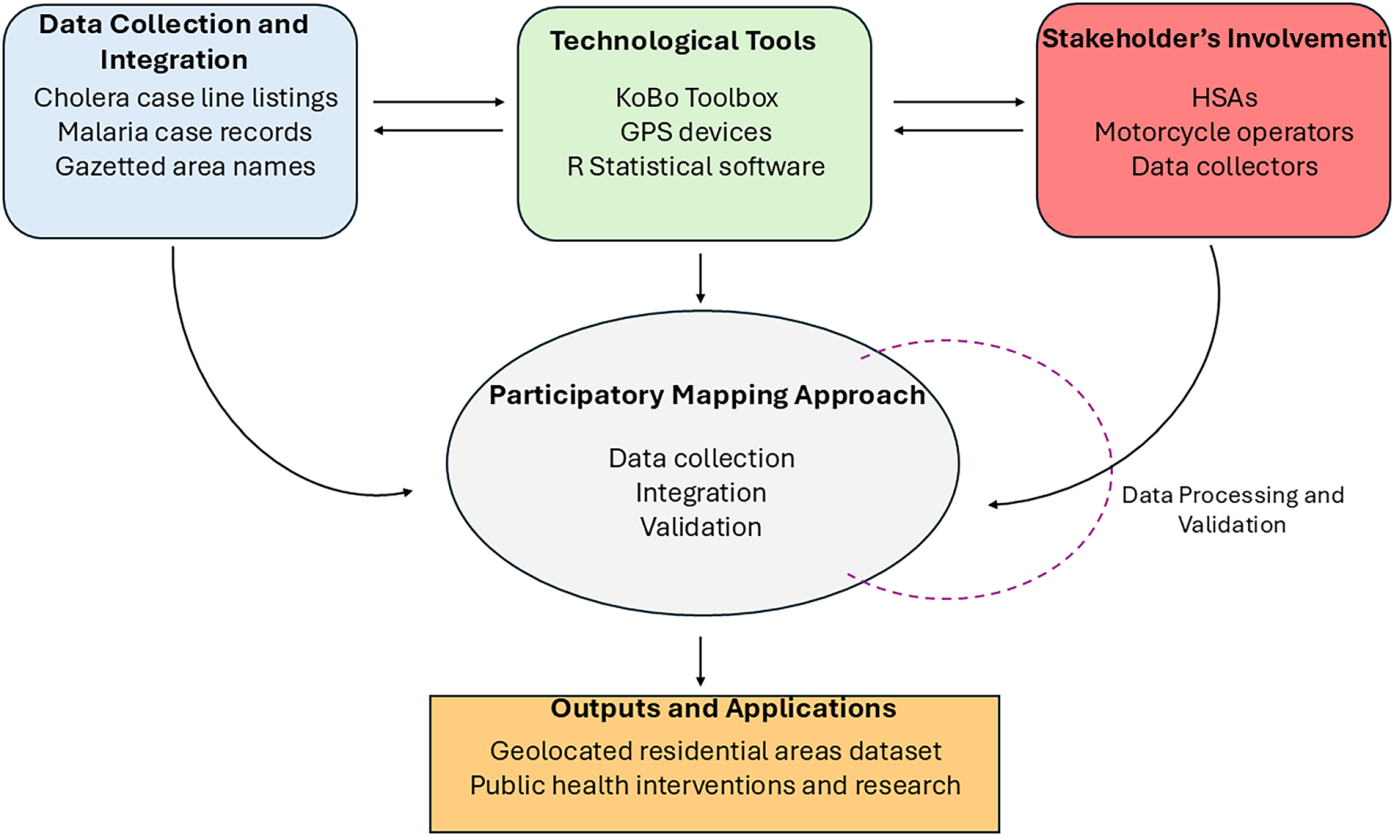
Schematic overview of study design and workflow, highlighting the integration of data  collection and integration (blue), technological tools (green), and stakeholder involvement (red) in participatory mapping approach (center). The data processing and validation loop (purple) represents the iterative nature of data verification and quality assurance. The workflow culminates in a comprehensive geospatial dataset for public health applications (orange).

To achieve this goal, we developed a comprehensive workflow that integrates various data sources, technological tools, and stakeholder involvement. Figure 1 provides a schematic overview of our study design and methodology, illustrating the interconnected steps in our data collection and processing approach.

As depicted in Fig. 1, our approach leverages multiple data sources, including cholera case line listings, gazetted names, and malaria case records. We utilised technological tools such as KoBo Toolbox for data collection and R software for data processing^25^. The workflow emphasizes the crucial role of stakeholder involvement, particularly the engagement of HSAs and local community members, in ensuring the accuracy and comprehensiveness of our dataset. Our validation process incorporated feedback from multiple stakeholders, creating an iterative approach to data refinement and quality assurance.

This study is timely, considering the recent cholera epidemic, the ongoing threats from climate change, rapidly growing informal settlements, and the potential for emerging and re-emerging epidemics – all of which require robust spatial-temporal analysis of disease patterns. By creating a comprehensive fine-scale location dataset through the process outlined in Fig. 1, we aim to enable more accurate identification of localized disease transmission patterns, crucial for effective public health interventions.

By advocating for the public sharing of this updated dataset through platforms like MASDAP^12^, we aim to enhance the comprehensiveness of public health research and interventions. This approach not only bridges existing data gaps but also provides valuable insights for refining public health strategies in Blantyre and similar resource-limited settings.

Through this study, we demonstrate the feasibility and importance of creating accurate, accessible geospatial data in challenging urban environments. Our findings have broad implications for public health planning, disease surveillance, and targeted intervention strategies, potentially serving as a model for similar efforts in other resource-constrained regions globally.

## Data and Methods

### Study area

**Fig. 2:**
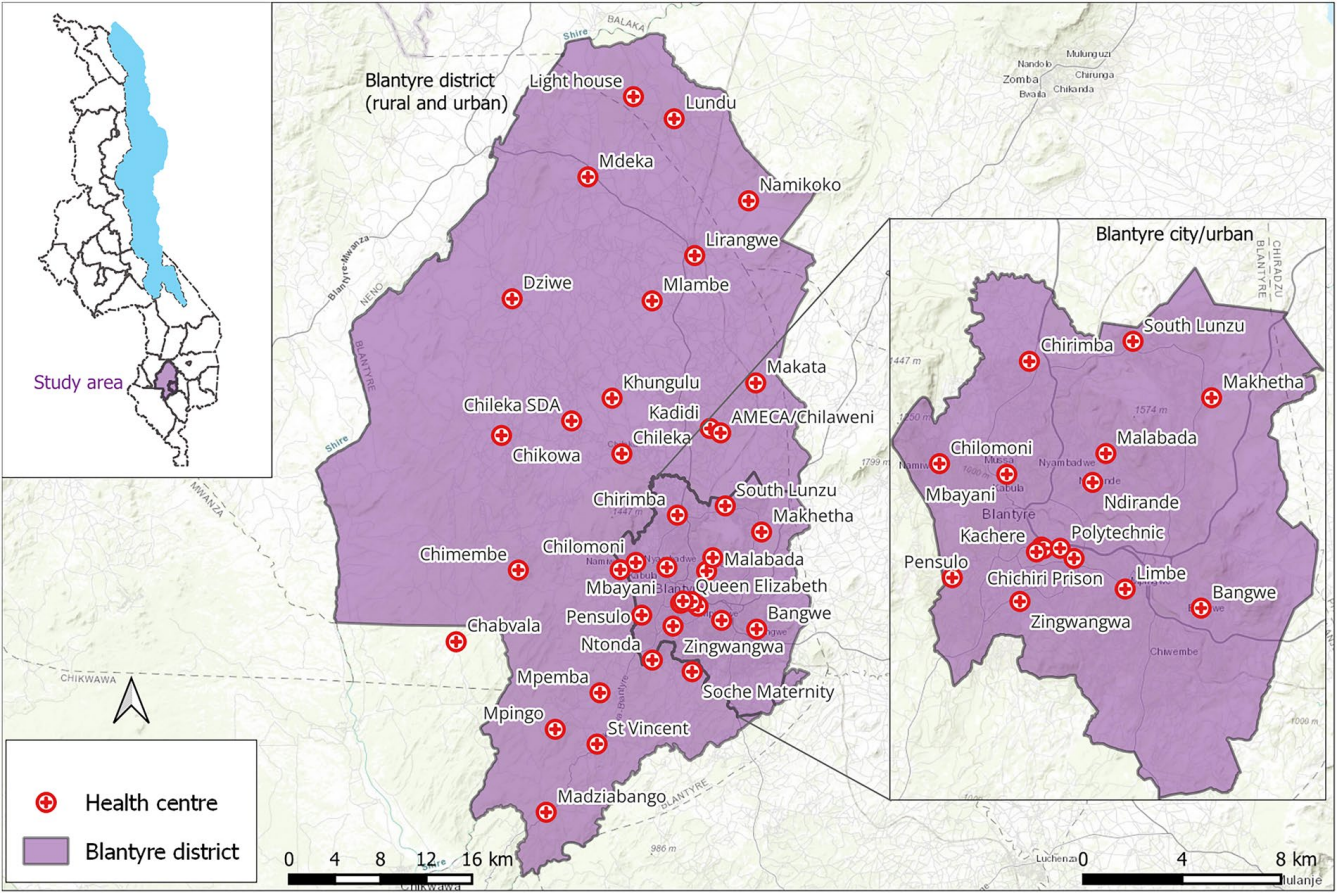
Map of the study area showing Blantyre city/urban within Blantyre district and the distribution of health centres to which patients report for care. Inset: Map of Malawi showing the location of Blantyre district.

Our study focused on Blantyre district. The district has 39 government-operated health facilities, with 16 in the city and 23 in rural areas. These health facilities served as key operational hubs for engaging with HSAs and planning our field mapping activities (Fig. 2). It is worth noting that in rural Blantyre, the population is sparsely distributed, with residential areas typically surrounded by farmland. Western Blantyre, in particular, has the lowest population density, and health facilities are more widely spread compared to other parts of the district. While this distribution may result in some underrepresentation of residential areas in western Blantyre, we mitigated this limitation by analyzing patient records from health facilities bordering western Blantyre that serve these communities, allowing us to capture residential areas whose residents seek healthcare outside district boundaries. Additionally, we consulted community leaders and village heads from western Blantyre to validate and supplement our residential area listings.

### Data collection and integration

**Fig. 3:**
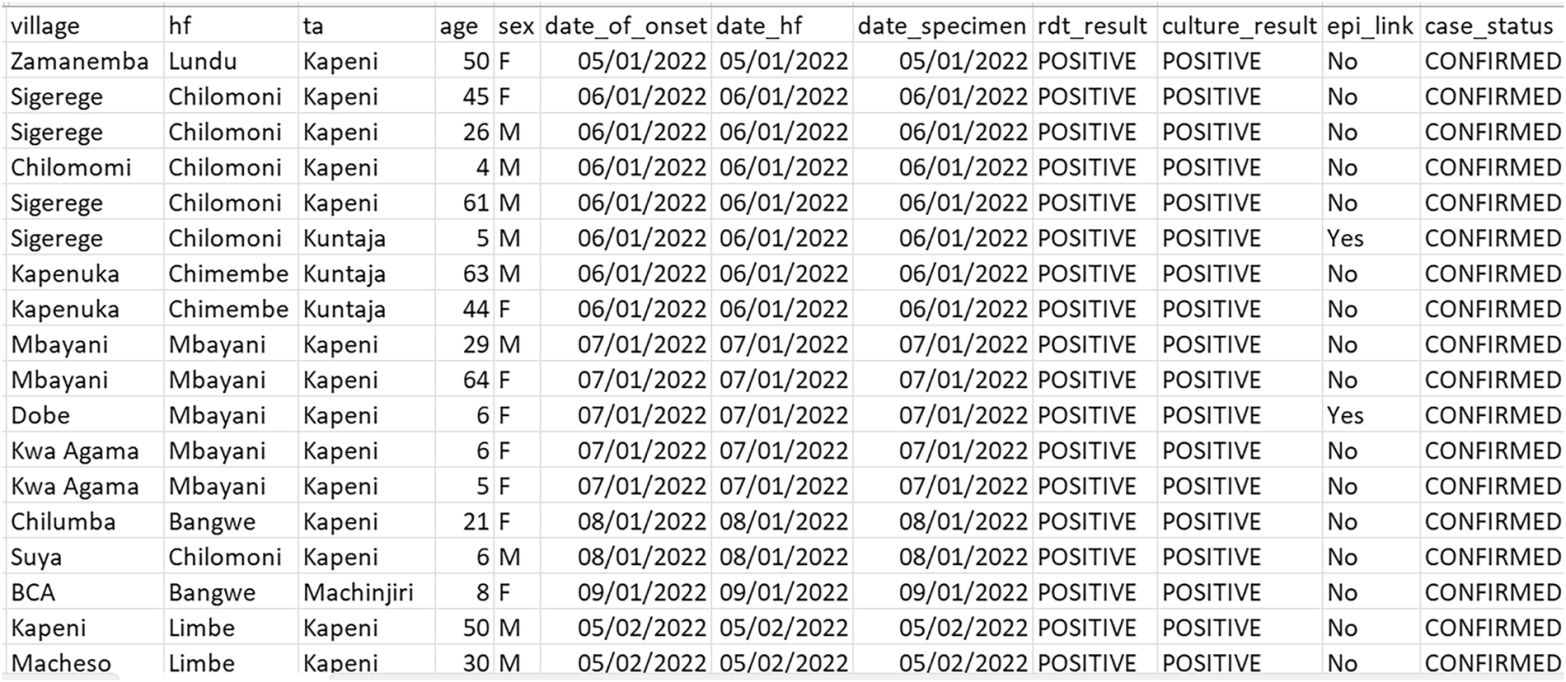
Sample snapshot of cholera records used for geolocating residential areas of cholera patients. The dataset includes patient identifiers (anonymized), reporting dates (onset of symptoms, health facility visit, and specimen collection), residential locations, and clinical details that informed our geolocation process. Patient identifiers have been obscured to protect privacy.

To establish a comprehensive list of residential area names, we compiled data from three primary sources namely, locations from recorded cholera cases, malaria cases and official names or residential areas gazetted by the government of Malawi. Cholera records formed our primary dataset given the recent outbreak. First, we obtained cholera line listing (example depicted in Fig. 3) from Blantyre District Health Office (DHO)^26^. We also included names of residential areas obtained from malaria records^27^. Malaria is an endemic disease in Malawi, and it is one of the most comprehensively documented diseases in local health facilities, providing additional residential area names that might not appear in cholera records. Furthermore, we collected gazetted names from Blantyre DHO, which are official residential area names listed by district councils in Malawi^28^. These datasets were combined using R Statistical Software version 4.3.1^25^ to create a unified database of names of residential areas in Blantyre district. As such, our consolidated dataset had a list of names of residential areas.

### Participatory mapping approach

We adopted a participatory mapping approach, an approach that recognizes the value of local knowledge and aims to collaborate with community members to contribute their unique expertise to the mapping process with the support of governmental institutions, non-governmental organizations (NGOs) or academic institutions^29,30^. We conducted five meetings with authorities from Blantyre DHO and MLW to encourage participation and ownership in the mapping process. These meetings facilitated discussions on the approach and goals of mapping.

We recruited and trained five data collectors: two research assistants from MLW and three Youth Mappers each from Malawi University of Business and Applied Sciences, University of Malawi and Malawi University of Science and Technology. Youth Mappers are a global community of independent university student groups that find common ground in their use of geospatial tools and platforms^31^. Through collaboration, they tackle authentic community challenges and empower positive change. We advertised the call for volunteer mappers through a WhatsApp group created for Malawi OpenStreetMap Community (details of this community can be accessed elsewhere^32^). These Mappers were selected based on their interest and enthusiasm for the project. The two research assistants were engaged based on their interest in the project and their high proficiency in the use of KOBO Toolbox. We organized a one-day training session, covering introduction to KoBo Toolbox, data collection methodologies, process of capturing geographical coordinate, and troubleshooting technical issues in the field.

We engaged HSAs to provide local knowledge and guidance within their working catchment areas. HSAs are community-based health workers who serve as contact points between the health system and communities in Malawi. They live and work within their assigned communities, conducting health surveillance, providing basic health services, and maintaining detailed knowledge of their catchment areas^33^. Their deep understanding of local geography, community structures, and residential patterns made them invaluable resources for this mapping exercise, as they could accurately identify and validate location names and boundaries that might not be apparent to outside observers.

Our data collection process began with preliminary preparation, where residential area names were printed and distributed to data collectors for planning purposes. We then created an online survey using KOBO Collect Toolbox (https://www.kobotoolbox.org/)^34^, deployable offline on smartphones. The survey captured data collector’s identity, associated health facility, residential area name, and geo-point coordinates of residential areas. We used the KoBoToolbox dashboard for real-time monitoring of progress, with access restricted to authorized personnel. Daily reviews of collected data were performed to identify and rectify errors and inconsistencies. We also implemented a feedback mechanism allowing data collectors to report issues, ask questions, and seek clarification to improve the data collection process.

**Fig. 4:**
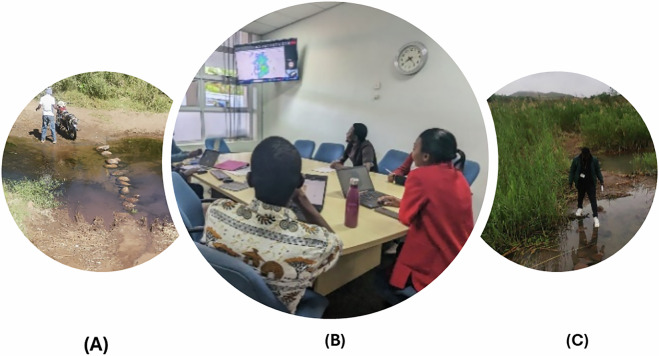
(**A**) Motorcycle operator, (**B**) Geospatial scientists monitoring and analysing collected data in real-time using the KoBoToolbox dashboard, and (**C**) data collector crossing a local river in Blantyre district.

The participatory mapping approach involved collaboration between data collectors, HSAs, motorcycle operators, Blantyre DHO, and MLW. Data collectors were responsible for gathering and recording spatial data on residential areas, HSAs guided data collectors to ensure comprehensive coverage, motorcycle operators and MLW provided transportation and logistical support enabling efficient data collection across a wider area, Blantyre DHO provided cholera line listing, gazetted residential areas and coordination with the HSAs. Figure 4 shows respective teams taking specific roles in ensuring efficient data collection across the district. This method aligns with recommendations from the United Nations Millennium Development Goals (MDGs) and the World Summit on the Information Society^35^.

### Data processing and validation

The mapping process resulted in a dataset containing latitude and longitude coordinates for 764 residential areas, with coordinates typically captured at an identifiable landmark within each residential area. These landmarks included community gathering points (e.g., village chiefs’ residences, community halls, marketplace centers), infrastructure features (e.g., health posts, communal water points), notable physical landmarks (e.g., distinctive rock formations), or central road junctions within the residential area. This data collection was conducted from July 3 to July 24, 2023, spanning 15 working days. Our data validation process ensured GPS precision of <5 meters for each coordinate and linked each coordinate to a specific data collector for traceability. We implemented data integrity constraints in the KoBo collect survey design and conducted visual assessment of collected geo-points using the KoBo Toolbox map element. Validation meetings were held with Blantyre DHO to verify mapped locations.

To ensure high geolocation accuracy, we implemented a comprehensive validation strategy. Beyond the technical precision of <5 meters provided by our GPS-enabled devices, we conducted a systematic accuracy assessment through multiple methods. First, we performed satellite imagery validation by comparing our collected coordinates against high-resolution imagery from Google Earth, evaluating consistency with visible settlement patterns and landmarks. Second, we cross-validated our coordinates against existing datasets in OpenStreetMap where available. When discrepancies occurred, we initiated a review process involving re-consultation with the relevant HSA. Finally, we conducted follow-up field visits to re-confirm coordinates.

**Fig. 5:**
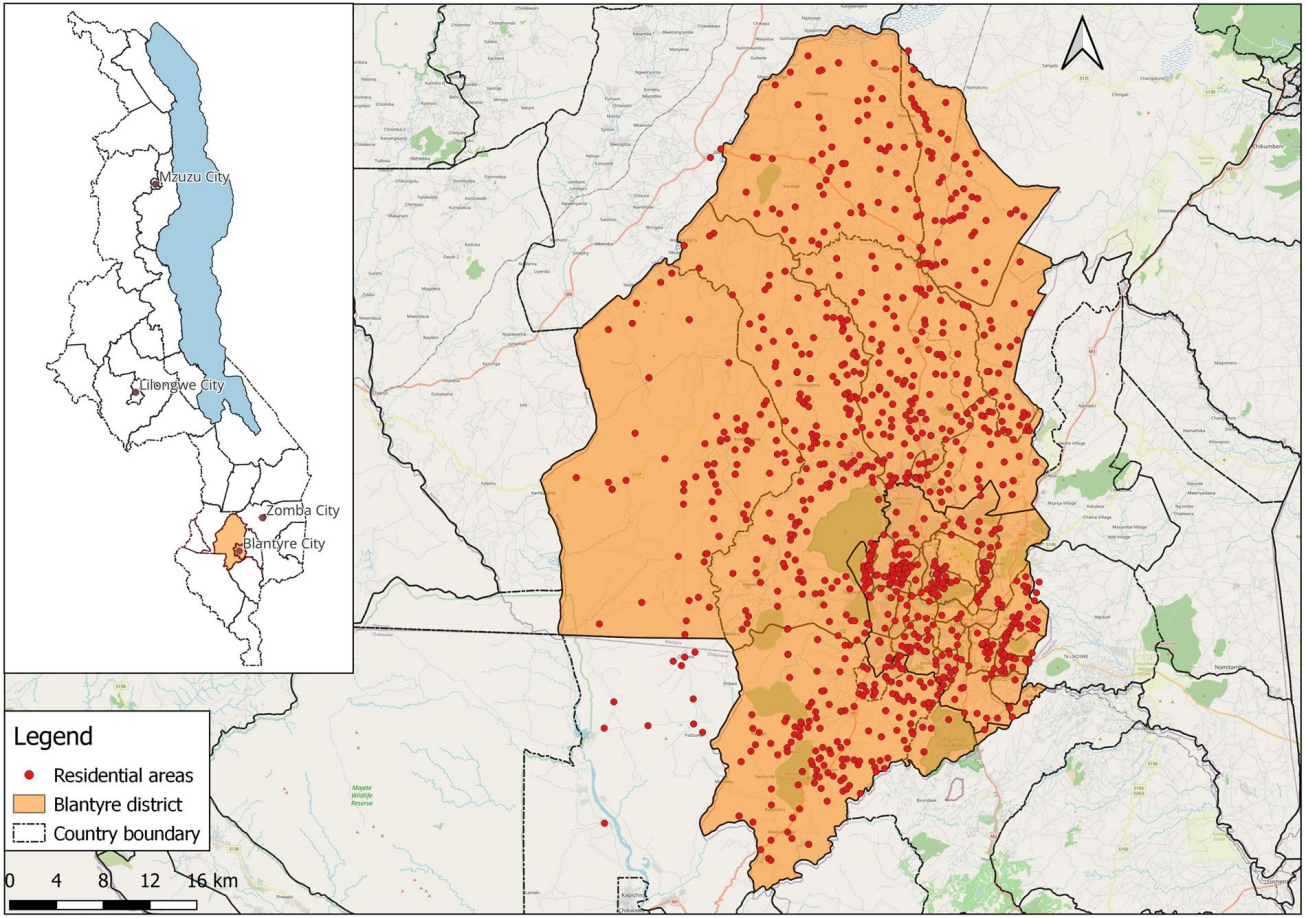
Map displaying the spatial distribution of geolocated residential areas in Blantyre district. The inset in the map of Malawi shows where Blantyre district is geographically placed and boarders with other districts as well. Blantyre district is subdivided into city and rural areas. These are further divided into Traditional Authorities (TA’s) or Wards. The red dots represent geolocated residential areas. Some residential areas geolocated outside Blantyre district were done intentionally as these areas indicated to have a good number of people accessing services in health centers in Blantyre district and will be used for further analysis.

Figure 5 provides a visual picture of residential areas in space. Data cleaning was performed using R Statistical Software version 4.3.1^25^, including spelling corrections and duplicate removal.

During data integration, we encountered multiple spelling variations and typographical errors in residential area names across different data sources. We addressed these inconsistencies through a standardization process that included manual verification with HSAs and local authorities to confirm matching locations. This approach enabled us to resolve spelling variations across 764 unique locations, ensuring data integrity while preserving local naming conventions.

### Cost analysis

The total cost of the mapping process was 2,005.69 USD. Costs were calculated based on actual spending converted from Malawian Kwacha (MWK) to USD using median exchange rates from July 3–24, 2023, the actual spending period. Table 1 provides a detailed breakdown of costs, including categories such as lunch allowances (based on predefined rates set by MLW), transportation, and airtime for communication and data syncing.

**Table 1:** Detailed cost breakdown of the mapping process.

Cost category	Items counted in costing	Amount ($)
Lunch allowance	MLW driver	81.34
	HSAs	216.91
	Data collectors	488.05
Transportation	Home to gathering point reimbursements	271.14
	Local movements within communities	892.22
Airtime	Communication and data syncing	56.03
**TOTAL**		**2005.69**

To contextualize the cost-effectiveness of our mapping initiative, we compared our expenditure of $2,005.69 for mapping 764 residential areas in Blantyre district with similar mapping initiatives. Our approach demonstrates significant cost savings, particularly when contrasted with conventional mapping efforts in the region.

Informal discussions with surveying professionals indicate that conventional mapping exercises in Malawi utilizing professional surveyors may incur significantly higher costs, ranging from $15,000 to $30,000 for comparable coverage. This translates to $15–25 per mapped location, requiring 30–40 days for completion. These figures underscore the potential for cost reduction through localized and participatory mapping approaches.

Our project’s cost per mapped location stands at approximately $2.60, which is lower than the per-location costs observed in conventional mapping exercises in Malawi. This efficiency highlights the feasibility and scalability of community-engaged mapping strategies in resource-constrained settings.

Our participatory mapping approach reduced costs primarily through leveraging local knowledge (eliminating need for extensive training), utilizing existing HSA networks (reducing recruitment costs), and employing simplified technologies that did not require specialized equipment. While direct comparisons must account for differences in geography, infrastructure, and project scope, our approach demonstrates significant cost advantages over conventional mapping methodologies while maintaining acceptable spatial accuracy (<5 meters).

### Timeliness

Time efficiency is a critical consideration for mapping initiatives supporting outbreak response. Our data collection was completed in 15 working days (July 3–24, 2023, excluding weekends), covering 764 locations across Blantyre district. This translates to approximately 51 locations mapped per day by our team of five data collectors. Put differently, each data collector recorded an average of 10 residential locations per day.

To contextualize this timeliness, conventional mapping approaches employing professional surveyors typically require 4–6 weeks for similar coverage. On the other hand, remote sensing approaches, while potentially faster for initial data acquisition, necessitate substantial post-processing time and ground verification. For instance, UNICEF’s application of GIS and remote sensing in Ethiopia and Madagascar highlighted the need for extensive post-processing and field validation, often resulting in a total time investment of 3–4 weeks^36^.

When incorporating timeliness into our cost-benefit analysis, our approach demonstrates significant advantages. Table 2 presents a comparative analysis of time, cost, and coverage metrics against conventional professional survey. Sustainability was assessed based on four key criteria. First, local capacity requirements - the extent to which the approach builds local expertise versus relying on external specialists. Second, ongoing resource needs - long-term costs for maintaining and updating the dataset. Third, community ownership - level of local stakeholder engagement and investment in the process, and fourth, scalability - ease of replication in similar setting. Our participatory approach achieved 'Medium' sustainability due to its reliance on existing HSA networks and community knowledge, while conventional surveys received 'Low' rating due to dependence on external expertise and higher ongoing costs.

**Table 2:** Comparison of mapping approaches by time, cost, and coverage metrics.

Mapping Approach	Time Required	Cost per Location/$	Coverage Rate	Accuracy/m	Sustainability
Our Participatory Approach	15 working days (51 locations/day)	2.55	59.5	<5	Medium
Conventional Professional Survey	30–40 working days (20 locations/day)	15–20	75–85	<2	Low

This temporal advantage is particularly valuable in outbreak contexts where rapid intervention is essential for controlling disease spread. The WHO’s outbreak response guidelines emphasize the importance of timely data collection and mapping to inform intervention strategies effectively. While specific timeframes may vary depending on the nature of the outbreak, the median time to detection in the WHO African Region has been reported as 8 days, with a median time to end of 77 days^37^. Our methodology’s completion timeframe of 15 working days aligns with this recommendation, enhancing its suitability for emergency public health applications.

## Data Records

The primary output of this study is a comprehensive geospatial dataset of residential areas in Blantyre district, Malawi. This dataset is publicly available through Zenodo (10.5281/zenodo.15487740)^38^ and is shared with the Blantyre DHO and other relevant public institutions for operational use in public health planning and interventions.

### Dataset structure and formats

The published dataset includes a comma-separated values (CSV) file containing the complete record of 764 geolocated residential areas, a GeoJSON file with identical information optimized for geospatial applications and supplementary documentation describing the data collection methodology and usage guidelines. Table 3 provides a complete data dictionary for dataset attributes.

**Table 3:** Data dictionary for Blantyre district residential areas dataset.

Attribute Name	Data Type	Description	Example Values
Location Name	String	Name of the residential area as used in health records	“Buleya”, “3 Ways”
Y	Decimal	Latitude coordinate in decimal degrees (WGS84)	15.78654
X	Decimal	Longitude coordinate in decimal degrees (WGS84)	35.02489
TA/Ward Name	String	Traditional Authority or Ward name	“TA Kapeni”, “Mbayani Ward”
Health Facility	String	Name of the reported health facility catchment area	“Zingwangwa”
Area Type	String	Classification as urban or rural	“Urban”, “Rural”
Date Mapped	Date	Date when coordinates were collected (YYYY-MM-DD)	“2023-07-15”

### Technical specifications

This geospatial data uses the WGS84 (EPSG:4326) coordinate reference system, which is compatible with most GIS software and web mapping applications. Each residential area is represented by a single point, typically captured at a central landmark within the settlement as described in the methods section. The dataset achieves a spatial accuracy of <5 meters for each coordinate, validated through the processes detailed in the Technical Validation section.

### Data access and usage

The dataset is provided under a Creative Commons Attribution 4.0 International License (CC BY 4.0), allowing for both academic and applied use with appropriate attribution. Users are encouraged to cite this publication when utilizing the dataset.

## Technical Validation

Extending beyond the initial data collection and processing methods, we implemented a comprehensive validation strategy. This validation process was through visual assessment of collected geo-points using the KoBoToolbox map element. This visual inspection allowed us to identify spatial outliers and anomalies that might not be immediately apparent in tabular data, providing an additional layer of quality control. We also organized a dedicated validation meeting with the Blantyre DHO. During this session, local health authorities reviewed the mapped residential area geo-points, drawing on their in-depth knowledge of the district. This stakeholder validation proved invaluable in verifying the accuracy of our dataset and identifying any discrepancies between recorded locations and local understanding.

We also conducted a thorough coverage analysis to understand the scope and limitations of our dataset. From the initial 1285 unique locations identified across our data sources, 803 were from cholera line listing, 395 from gazetted district council names, and 87 from malaria records. We successfully geolocated 764 locations, achieving a 59.5% coverage rate.

Most of the unmapped locations came from cholera surveillance line listing, particularly where individual residence names or non-standardized location descriptions were used instead of established community names. This highlights a critical need to standardize geographical information capture during health facility visits. This analysis not only highlighted the extent of our mapping efforts but also helped us identify areas for potential future expansion of the dataset.

Following the stakeholder validation, we undertook an additional round of data refinement. This process involved standardizing location names, resolving discrepancies between informal names used in health records and official gazetted names, and addressing instances of multiple spellings for the same location. This refinement phase was crucial in enhancing the consistency and usability of our dataset.

As a final step, we performed a comprehensive quality assurance review. This ensured that all validation processes were properly documented and that any changes made during the refinement process were justified and traceable, maintaining the integrity of our data.

While our dataset provides extensive coverage of residential areas recorded in official sources, we acknowledge certain limitations in capturing the complete geographical hierarchy of locations. For instance, a single area might be recorded at different administrative levels (e.g., a location recorded variously as “Pankhumba,” “Ngumbe,” or “Chileka” - representing different levels of geographical detail for the same area). This variation in recording practices can pose challenges when linking disease cases to specific locations. To address the challenge of capturing the complete geographical hierarchy of locations, we introduced ‘TA/Ward Name’ and ‘Area Type’ attribute that explicitly categorizes each residential area. This classification helps users understand the nature of each location and its relative position in the settlement hierarchy, reducing confusion when the same general area might be recorded at different administrative levels.

This limitation, inherent to our data sources, should be considered when utilizing the dataset for analysis or decision-making purposes. Despite this, the multiple layers of validation and quality control measures implemented contribute significantly to the overall technical rigor and reliability of our geolocated residential areas dataset for Blantyre district.

### Data quality assessment

To ensure transparency and reliability of our dataset, we conducted a structured evaluation of data quality and systematically assessed potential biases introduced by our methodology. This evaluation examines multiple dimensions of data quality, including coverage, representativeness, spatial accuracy, and completeness.

### Comparative coverage analysis

Our 59.5% coverage aligns with previous findings showing that community-led mapping and community health workers (CHW) delivered service programs in resource-limited settings typically achieve coverage in the 50–60% range (e.g., Mali CHW malnutrition coverage increased to ≈ 57%)^39^, suggesting our hybrid model is comparably effective.

In evaluating coverage sufficiency for disease transmission prevention, the European Centre for Disease Prevention and Control (ECDC) recommends that targeted interventions in high-risk areas can be more effective than uniform coverage across all areas, especially when resources are constrained^40^. This strategy supports the notion that achieving 59.5% coverage in high-risk zones can significantly enhance intervention efficacy. Our dataset’s strategic coverage pattern aligns with this approach, reinforcing its appropriateness for supporting cholera intervention strategies in Blantyre district.

### Coverage and representativeness

Our analysis of dataset coverage revealed important patterns across different area types. Table 4 summarizes the distribution of mapped locations across settlement categories.

**Table 4:** Distribution of mapped locations by area type.

Area Type	Number of Locations	Percentage
Urban	272	35.6
Rural	492	64.4
**Total**	**764**	**100**

The observed disparity in our mapping coverage of 64.4% in rural areas versus 35.6% in urban areas can be attributed to several factors inherent to urban environments. Urban areas often present challenges such as complex infrastructure, high population density, and presence of informal settlements, which can impede data collection efforts. Informal settlements, in particular, are frequently underrepresented in official records and maps, leading to gaps in data coverage^41^. These factors collectively contribute to the lower representation of urban areas in our dataset.

### Potential biases and limitations

We identified several potential biases in our data collection approach. Our reliance on health facility records likely underrepresented communities with limited healthcare access. Areas with lower healthcare utilization rates may be systematically underrepresented in our dataset, particularly affecting remote rural communities. Data collection in some areas was hampered by geographical barriers (e.g., poor road infrastructure), potentially leading to underrepresentation of hard-to-reach communities. As visible in Fig. 5, there are noticeable gaps in the distribution of mapped residential areas, particularly in western and southern rural regions, which correlates with accessibility challenges documented during data collection.

The higher concentration of mapped locations in rural areas compared to urban areas indicates potential sampling bias toward areas with higher disease incidence or better disease reporting systems created by our primary reliance on cholera records. Communities with fewer reported cholera cases might be underrepresented despite our supplementation with malaria records and gazetted names.

### Quality metrics

To quantify data quality, we evaluated several key metrics. We assessed dataset completeness by comparing our mapped locations to those identified in our combined source datasets. As noted earlier, we achieved 59.5% coverage (764 of 1285 unique locations), with higher completeness in rural areas (64.4%) compared to urban areas (35.6%).

We evaluated naming consistency through stakeholder validation meetings, identifying and resolving locations with multiple spelling variations or conflicting names. This standardization process significantly improved dataset usability.

Our data collection occurred in July 2023, ensuring temporal relevance to the 2022-2023 cholera outbreak. However, rapidly changing settlement patterns, particularly in urban areas, may reduce dataset accuracy over time.

### Implications and recommendations

The identified biases have several implications for dataset applications. When using this dataset for disease surveillance or intervention planning, users should be aware of potential underrepresentation of hard-to-reach residential areas, which may affect analysis conclusions.

To mitigate these biases in future data collection efforts, we recommend incorporating additional data sources beyond health facility records, such as community registries to improve coverage of areas with limited healthcare access. Employing stratified sampling approaches would ensure proportional representation across settlement types. We also recommend implementing regular update mechanisms to capture newly established settlements, particularly in rapidly developing urban areas, and triangulating coordinates with multiple sources to improve spatial accuracy in areas with complex settlement patterns.

Despite these limitations, our dataset represents a significant improvement over previously available geospatial data for Blantyre district, especially for public health applications related to the cholera outbreak response.

## Scalability and Transferability

The participatory mapping methodology developed in this study has significant potential for scalability beyond Blantyre district to other regions in Malawi and similar settings globally. Several features of our approach make it amenable to scaling. Low technical requirements, minimal training needs, reliance on existing health system structures, and cost-effectiveness. The approach represents an economically viable solution for resource-constrained settings.

Scaling this methodology to other districts within Malawi would be relatively straightforward due to similar health system structures. The HSA network exists throughout the country, providing a ready workforce with local knowledge that could be mobilized using the same approach. The KoBo Toolbox platform used for data collection is freely available and can accommodate larger datasets without licensing costs. Our R scripts for data processing are open-source and adaptable to different datasets with minimal modification.

For expansion to other countries, the methodology would require adaptation to local health system structures. Countries with community health worker programs similar to Malawi’s HSA system such as Rwanda’s Community Health Workers, Ethiopia’s Health Extension Workers, or Zambia’s Community Health Assistants could adopt this approach with minimal modifications. The essential requirement is access to frontline health workers with detailed knowledge of their catchment areas.

However, several potential barriers to scalability should be considered. First, administrative and stakeholder buy-in may vary across regions. Our success in Blantyre was partly due to strong relationships with the DHO and existing research partnerships. Building similar relationships in new areas would require time and diplomatic engagement. Second, geopolitical factors may complicate implementation in some settings. Areas with territorial disputes, security concerns, or highly mobile populations would present additional challenges for mapping teams. Third, differing settlement patterns and population densities would affect implementation efficiency. While our approach worked well in Blantyre’s mixed urban-rural environment, extremely sparse rural settlements or dense informal urban areas might require methodological adaptations. Finally, integration with existing HIS varies across countries. Where electronic health records or health management information systems are already in place, additional steps might be needed to ensure compatibility and avoid duplication.

Despite these challenges, this methodology offers a practical framework that can be adapted to diverse settings. For optimal scaling, we recommend a phased approach beginning with pilot implementations in new areas, followed by adaptations based on local feedback before full-scale deployment. The participatory nature of our methodology means that it inherently incorporates mechanisms for local adaptation, as community health workers and local stakeholders contribute their expertise to the mapping process.

Future research should focus on developing standardized protocols for adapting this methodology to different settings, particularly exploring integration with other HIS and tailoring to various settlement patterns. Additionally, longitudinal studies evaluating the sustainability of mapping efforts and mechanisms for regular updates would strengthen the long-term value of this approach.

## Data Availability

The data cleaning and processing for this study were performed using R Statistical Software version 4.3.1. We developed a custom R script to facilitate the cleaning and standardization of location data. This script, along with all associated codes used in the data processing pipeline, has been made publicly available without any access restrictions. The code repository is hosted on the MLW geospatial technical team’s GitHub platform, which can be accessed at https://github.com/MLW-geospatial-technical-team/Blantyre_location_dataset/blob/main/cleaned_villages%20for%20manuscript.R^42^. Our decision to use GitHub, an AI-powered developer platform, ensures that the code is easily accessible, version-controlled, and can be collaboratively improved. The repository includes not only the main data cleaning script but also any additional functions or utilities developed for this project. We have also included detailed comments within the code to explain the purpose and functionality of each section, making it easier for other researchers to understand and potentially adopt our methods for their own studies. In addition to the R script, we have provided a README file in the repository that outlines the system requirements, necessary R packages, and step-by-step instructions for running the code. This file also includes information on any specific variables or parameters that users might need to modify when applying the code to their own datasets. We encourage researchers and data scientists to explore, use, and build upon our code. By making our code publicly available, we aim to promote transparency in our data processing methods and facilitate reproducibility of our results. If users encounter any issues or have suggestions for improvements, they can utilize GitHub’s issue tracker or submit pull requests to contribute to the ongoing development of this resource.
